# Movement smoothness during a functional mobility task in subjects with Parkinson’s disease and freezing of gait – an analysis using inertial measurement units

**DOI:** 10.1186/s12984-019-0579-8

**Published:** 2019-09-05

**Authors:** Camila Pinto, Clarissa Pedrini Schuch, Gustavo Balbinot, Ana Paula Salazar, Ewald Max Hennig, Ana Francisca Rozin Kleiner, Aline Souza Pagnussat

**Affiliations:** 1 Rehabilitation Sciences Graduate Program, Universidade Federal de Ciências da Saúde de Porto Alegre (UFCSPA), 245 Sarmento Leite Street, Porto Alegre, RS 90050170 Brazil; 2 Movement Analysis and Rehabilitation Laboratory, Universidade Federal de Ciências da Saúde de Porto Alegre (UFCSPA), Porto Alegre, RS Brazil; 3 Brain Institute, Universidade Federal do Rio Grande do Norte (UFRN), Natal, RN Brazil; 4 Institute of Health and Biomedical Innovation, Queensland University of Technology, Brisbane, QLD Australia; 5 Department of Physiotherapy, Universidade Federal de São Carlos (UFSCar), São Carlos, SP Brazil

**Keywords:** Parkinson’s disease, Mobility limitation, Movement disorders

## Abstract

**Background:**

Impairments of functional mobility may affect locomotion and quality of life in subjects with Parkinson’s disease (PD). Movement smoothness measurements, such as the spectral arc length (SPARC), are novel approaches to quantify movement quality. Previous studies analyzed SPARC in simple walking conditions. However, SPARC outcomes during functional mobility tasks in subjects with PD and freezing of gait (FOG) were never investigated. This study aimed to analyze SPARC during the Timed Up and Go (TUG) test in individuals with PD and FOG.

**Methods:**

Thirty-one participants with PD and FOG and six healthy controls were included. SPARC during TUG test was calculated for linear and angular accelerations using an inertial measurement unit system. SPARC data were correlated with clinical parameters: motor section of the Unified Parkinson’s Disease Rating Scale, Hoehn & Yahr scale, Freezing of Gait Questionnaire, and TUG test.

**Results:**

We reported lower SPARC values (reduced smoothness) during the entire TUG test, turn and stand to sit in subjects with PD and FOG, compared to healthy controls. Unlike healthy controls, individuals with PD and FOG displayed a broad spectral range that encompassed several dominant frequencies. SPARC metrics also correlated with all the above-mentioned clinical parameters.

**Conclusion:**

SPARC values provide valid and relevant clinical data about movement quality (e.g., smoothness) of subjects with PD and FOG during a functional mobility test.

**Electronic supplementary material:**

The online version of this article (10.1186/s12984-019-0579-8) contains supplementary material, which is available to authorized users.

## Background

Parkinson’s disease (PD) frequently affects mobility, postural transitions and balance, and compromises activities of daily living [1, 2]. Up to 75% of subjects with PD may present freezing of gait (FOG) [3]. FOG is characterized by sudden, brief episodes of inability to produce forward stepping, which occurs mostly during gait initiation or while turning. Functional activities in daily living involve approaching obstacles, turning, sitting or standing. All of these features are particularly challenging for subjects with PD and FOG. FOG is related to risk of falling [4, 5] and occurs mainly during the off-medication phase [6, 7]. Movement analysis during functional tasks could help to quantify FOG events and movement quality, e.g. smoothness, to unveil an associative or causal link between these parameters. Such information may be very useful for improving fall prevention programs associated with PD and FOG [1].

In clinical settings, one of the most common tests to assess functional mobility is the Timed up and Go (TUG). This test involves tasks commonly used in daily lives and includes turns, brakes, sit-to-stand and stand-to-sit movements. All these essential functional movements are challenging for individuals with PD and FOG. TUG is also used to monitor risk of fall in frail elderly and subjects with PD [8, 9]. Aligned with the increasing use of wireless technologies an instrumented version of the TUG test, also known as the iTUG test, has become quite popular [10, 11].

The iTUG test uses a wearable sensor built-in with Inertial Measurement Units (IMUs) - composed of sensors, such as accelerometers and gyroscopes. These sensors can quantify movement in an ecological context [12], which facilitate the measurement of several parameters, such as movement smoothness. Smoothness is defined as the amount of trajectory or velocity adjustments during a specific movement, reflects movement intermittency and is related to movement coordination [13, 14]. Smoothness in non-pathological walking is continual or non-intermittent, representing a rhythmic and coordinated pattern of gait [15]. Assessments of smoothness by means of spectral analysis have been used in several contexts to provide quantification of movement quality [16–18]. However, this measure has been underexplored in individuals with PD during functional mobility tests.

Several smoothness metrics have been used in recent years, such as the number of peaks, dimensionless and log dimensionless jerk, and spectral arc length (SPARC) measures (reviewed in [13]). A smoothness measure is considered useful if it is dimensionless, consistent, sensitive and robust [13]. Expert recommendations about smoothness measurement using acceleration and recorded by accelerometers suggest the use of SPARC over jerk metrics [13]. Although both of these metrics show reduced effects of the movement amplitude and duration, SPARC is estimated to be 10 times less susceptible to signal-to-noise ratio artifacts [13]. This aspect is of particular importance when analyzing data from accelerometers, which are more prone to noise when compared to lab systems for recording kinematic data, for example [13, 19]. Point-to-point reaching tasks are the most studied in the literature and have SPARC scores of ≈ − 1.6 [13]. SPARC, was previously used to describe PD-related gait impairments using tri-axial accelerometer data, and showed values of ≈ − 5.5 (Control), − 5.8 (PD-ON) and − 6.3 (PD-off) [19]. Additionally, normative values for SPARC are needed for the iTUG task.

Thus, monitoring and quantifying movement smoothness during functional mobility tasks are quite important. The TUG test would be a suitable test to address this issue because it requires highly relevant skill components of mobility [20]. The literature shows evidence that freezers present higher variability and less consistency of gait compared to non-freezers [21, 22]. Then, we decided to investigate movement smoothness during the TUG test only in subjects with PD and FOG in the off-medication phase. We chose to evaluate participants in the off-medication phase in order to avoid the effects of levodopa on FOG [23]. Also, we aimed to verify correlations between SPARC data and clinical parameters. We hypothesized that subjects with PD and FOG would present reduced smoothness (SPARC) [24], which would be correlated with clinical parameters.

## Methods

### Participants and procedures

This is a cross-sectional study approved by the Ethics Research Committee of the Federal University of Health Science of Porto Alegre (protocol n.: 1.333.131). Written informed consent was obtained from all participants. We followed the Strengthening the Reporting of Observational Studies in Epidemiology (STROBE) [25] checklist. To be considered as eligible, participants must have received diagnosis of idiopathic PD (according to the London Brain Bank Criteria) [26]. Subjects aged between 50 and 85 years who were able to walk at least eight meters with or without walking devices, presenting FOG episodes (as verified by the Freezing of Gait Questionnaire - FOG-Q) and with a minimum score of 20 in the Mini Mental State Examination (MMSE) were included. Subjects were excluded if they had previous history of musculoskeletal or neurological disorders or other clinical conditions that induced visible gait abnormalities, report of peripheral neuropathy and presence of deep brain stimulation devices. Healthy controls volunteers were included only as a reference group (male or female, aged between 50 and 80 years and with no history of musculoskeletal or neurological disorders).

At the first visit, subjects with PD performed the MMSE, motor part of the Unified Parkinson’s Disease Rating Scale (UPDRS III), Hoehn & Yahr scale (H&Y) and FOG-Q during off-medication phase. At the second visit, subjects were instructed to perform the TUG test, also in the off-medication state. The off-medication phase was defined according to each participant’s drug regimen. At the prescribed time of medication intake, the medication was not administered, and the participant was instructed to wait for one hour. After one hour, the testing was initiated. If the researchers noticed subjects were still in “on-phase” or “partially off state”, they waited until a subjective “off state” to start the test.

**Fig. 1:**
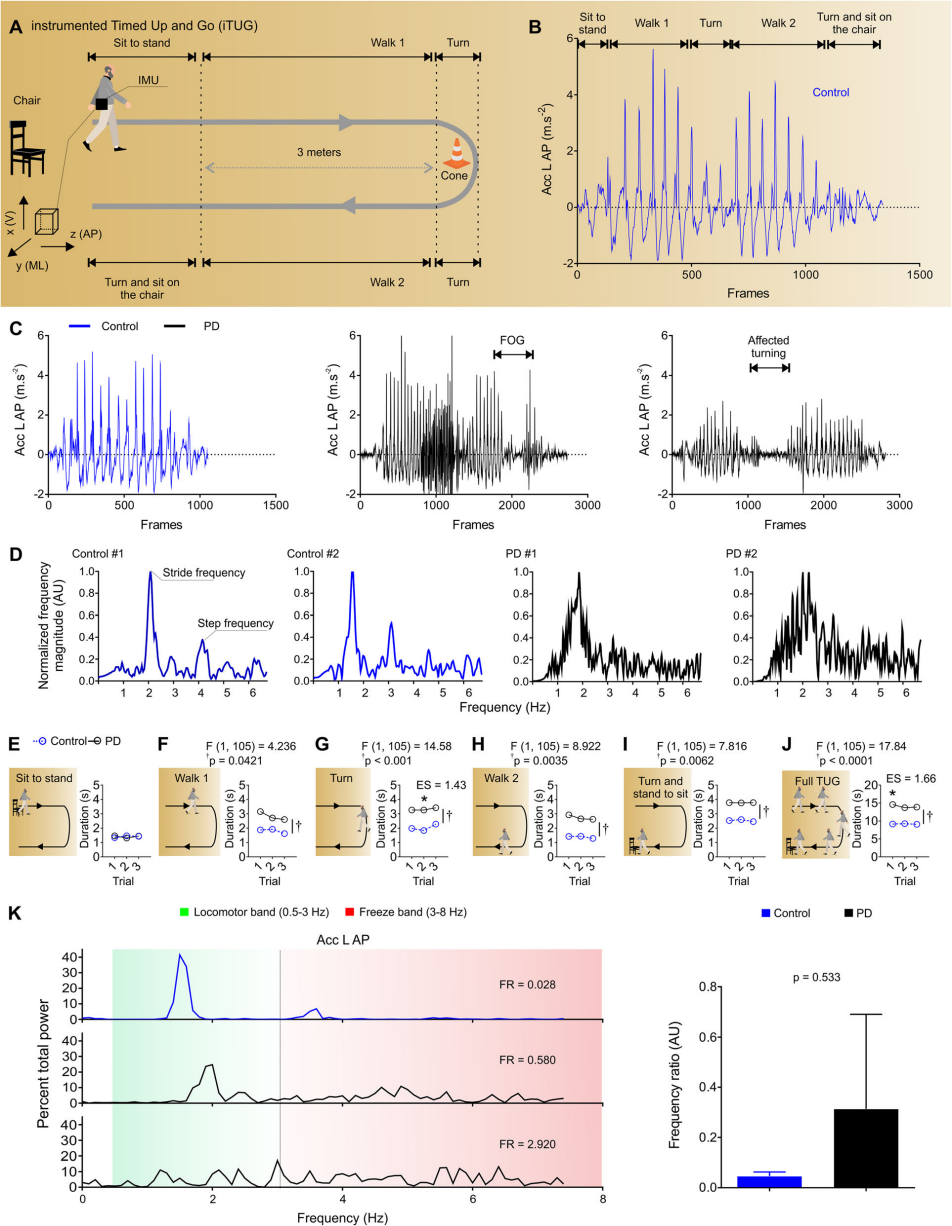
Instrumented Timed Up and Go test and representative spectral-domain of healthy controls and subjects with PD. **a** Instrumented Timed Up and Go test (iTUG). **b** Representative anteroposterior acceleration profile (Acc L AP) during the TUG test for a healthy subject. **c** Acc L AP of a healthy control (blue) and two subjects with PD (black) - note the FOG event and affected turning. **d** Representative spectral magnitudes of Acc L AP for two healthy controls and two individuals with PD during TUG trials. Note the well-defined magnitude peaks in the frequency domain in trials of control compared with trials of subjects with PD, which show a broader range of dominant frequencies and SPARC (area under the curve). **e-j** TUG phases were identified using yaw and pitch angles, the following TUG durations are described: sit to stand, walk 1, turn, walk 2, turn and stand to sit and full TUG. **k** Frequency ratio (FR) of a healthy subject (blue) and two subjects with PD (black), note the predominance of frequency components in the locomotor (green; 0.5–3 Hz) and freeze (red; 3–8 Hz) bands [1]. IMU: Inertial Measurement Unit; FOG: Freezing of Gait. FR: frequency ratio. Data are from representative subjects in **b-d** and **k** (left panel); and mean ± confidence interval 95% in **k** (right panel) and Mean in **e-j**; n_Control_ = 6; n_PD_ = 31; *p < 0.05

TUG was recorded using a Bluetooth-compatible commercial IMU system (BTS G-Walk BTS Bioengineering Corporation, Italy). This IMU System shows excellent reliability, accuracy and precision in quantifying total TUG test duration in patients with PD [27]. IMU was attached to the participants’ waist with a semi-elastic belt at L4-L5 height. All participants were wearing regular footwear and performed TUG test without walking devices. They were asked to rise from sitting on a standard chair, walk 3 m at self-selected speed, turn around a cone, walk back to the chair, and sit down (Fig. 1a). The test began when the evaluator said “go” and ended when the subject sat down with the back resting. Acceleration range was set to ±8 g, gyroscope range to ±250^o^/s and sampling rate of 100 Hz. The device included a triaxial accelerometer and a triaxial gyroscope. Signals acquired were linear (Acc L) and angular acceleration (Acc A) axes: vertical (V), mediolateral (ML) and anteroposterior (AP). Raw acceleration data were extracted using the G-sensor® software and exported in txt format. Participants performed the TUG test three times, and the total time in seconds was recorded. The average of three trials was used in the analysis.

### Data analysis

Signal processing was performed by using LabVIEW® (National Instruments, USA, v.18.0) custom software routines. Linear and angular data were considered when the mean of a moving window of 10 frames was greater than three times SD of the initial noise (100 frames window). We used mean subtractions to remove the direct current (DC) components from raw acceleration data and whenever signal manipulations caused drifting of the signal. Removing DC and drifting is important when processing acceleration measured by accelerometers, especially to remove the large DC component in the spectrum (accelerometers also pick up gravity) [13]. Subsequently, high frequencies not involved in the TUG test were removed when applying the limits of integration (lower bound = 0; upper bound = 10 Hz). SPARC calculation was adapted for the TUG test from the method described by Balasubramanian et al. 2015 [13]. We calculated the SPARC from each trial and the average SPARC from three TUG trials through the following formula:

1 $$ SPARC=-{\int}_0^{10}\sqrt{{\left(\frac{1}{10}\right)}^2+{\left(\frac{normPSD(w)}{dw}\right)}^2 dw} $$

where, 0 Hz and 10 Hz are the limits of integration, normPSD is the normalized power spectrum density (PSD) and dw is an infinitesimal amount of PSD frequency.

See Additional file 1 for detailed description of the algorithm used for SPARC calculation. For Acc L total and Acc A total we used the signal manipulations and equations proposed by *Beck* et al. *2018* [19]*.* Importantly, spectral analysis metrics follow the assumption that less smooth movements are more complex in terms of their frequency composition [17]. Therefore, lower SPARC values indicate less movement smoothness. SPARC was calculated for full TUG and also for separated phases such as (i) sit-to-stand, (ii) walk 1, (iii) turn, (iv) walk 2, (v) turn and stand-to-sit. Turns were identified from the yaw axis and sit-to-stand and stand-to-sit from the pitch axis. In order to increase FFT resolution and power of the dominant frequencies present in each TUG phase, we artificially increased data segments of each TUG phase by a factor of 4 (smaller TUG phases: sit to stand, walk 1, turn, walk 2 and stand to sit) and 2 (full TUG). This substantially increased SPARC sensitivity in addition to the zero padding procedures.

### Freeze detection

We did not have a video recording to annotate FOG events, thus, we used mathematical approaches to detect them. To automatically estimate the total amount of freeze-like behavior during the iTUG task, we opted to use simple methods – i.e. non-dependent of synchronized video analysis. Although machine learning methods are a better standard for freeze detection, it is necessary to visualize a freeze detection from a synchronized video recording, and to train the computer algorithm to detect further freeze events throughout the entire record (reviewed in [28, 29]). Here, we used a simple methodology that does not require video recordings: the Frequency Ratio (FR; Mancini et al., 2012 [30]). Briefly, this method calculates the square of the total power in the 3–8 Hz band (freeze band), divided by the square of the total power in the 0.5–3 Hz band (locomotor band). FR values close to zero indicate healthy gait, close to ≈0.3 PD gait (no freezing) and to ≈2 PD gait (with freezing). Importantly, while this method presents the best results when using the antero-posterior acceleration signals from accelerometers placed at the shank, here we used a waist mounted accelerometer.

### Statistics

Sample size was determined based on a previous study [19], adopting 90% power and alpha value of 0.05 to detect a mean difference of 1.57 with the standard deviation of 0.79 in SPARC total acceleration. The enrollment of 6 participants in each group (PD and healthy controls) was defined. We also determined the sample size necessary for correlations between questionnaires and SPARC metrics for PD group. We adopted a power of 95% and an alpha value of 0.05. We used the effect size (r) of 0.65 considering the correlation between UPDRS III and SPARC total acceleration [19]. A sample size of 20 participants was defined for PD group correlations.

A two-way ANOVA was used to compare groups and the three TUG trials. Sidak-correction was used for correction of multiple comparisons when appropriate. A correlation analysis, Pearson Moment Product, was performed between the clinical scores (UPDRS-III, H&Y, FOG-Q) and quantitative metrics to determine if a significant relationship existed. To minimize the bias of unbalanced groups, we used a Type II instead of Type III sum of squares for all analyses as described elsewhere [31, 32]. Statistical analysis was performed using the statistical software IBM SPSS version 25. Correlation maps were created using LabVIEW® 8.5. G-Power 3.0 software was used to calculate effect size, which was classified according to Cohen as small (0.2), moderate (0.5), and large (0.8). An effect size > 0.4 is considered clinically relevant [33]. Data were expressed as mean and 95% confidence interval, significance level was set at α < 0.05.

## Results

Thirty-one subjects with PD and FOG and six healthy controls were included (Table 1). We calculated the retrospective power considering the mean difference found between groups for SPARC total acceleration (mean = 2.11, standard deviation = 1.9). Adopting an alpha value of 0.05, we achieved a power of approximately 50%. The anteroposterior linear acceleration profile (Acc L AP) showed clear positive and negative peaks, reflecting the oscillatory breaking and propulsion forces applied on the ground during walking (Fig. 1b). Subjects with PD presented decreased acceleration magnitudes and slower turns when compared to healthy controls (Fig. 1c). Healthy controls showed well-defined frequency peaks (around ≈ 2 Hz and ≈ 4 Hz) that reflect the oscillatory nature of both stride and step cycles, respectively. On the other hand, subjects with PD showed a lack of well-defined frequencies, a broader range of frequencies and area under the curve (related to SPARC calculation; Fig. 1d). A two-way ANOVA revealed that PD participants spent greater time on walk 1, turn, walk 2, turn and stand-to-sit across trials compared to healthy controls (*p* < 0.05, Fig. 1e-i). Overall, PD subjects spent more time to complete the full TUG test compared to control subjects (*p* < 0.05, Fig. 1j).

**Table 1:** Demographic characteristics

	Subjects with PD	Healthy Controls
	*n* = 31	*n* = 6
Gender (F/M)	9/22	0/6
Age (years)	64.70 (61.28–68.13)	68 .33 (61.44–75.22)
body mass (kg)	76.09 (68.96–83.23)	77.63 (69.90–85.35)
Height (cm)	163 (159–167)	171 (161–180)
Time of disease (years)	9.29 (7.47–11.11)	–
MMSE	26.61 (25–27)	–
FOG-Q	14.67 (13–16)	–
H&Y off		–
1 / 2 / 2.5	1 / 4 / 6	
3 / 4	11 / 8	
5	1	
UPDRS III off	24.64 (21–27)	–

Data are mean and 95% confidence interval

Abbreviations. *PD* Parkinson Disease, *MMSE* Mini Mental State Examination, *FOG-Q* Freezing of Gait Questionnaire, *H&Y off* Hoehn & Yahr scale during off-levodopa phase, *UPDRS III off* motor part of the Unified Parkinson’s Disease Rating Scale during off-levodopa phase

SPARC values and time elapsed during TUG are shown in Table 2. All SPARC values were significantly lower in subjects with PD compared to healthy controls (*p* < 0.05), except for Acc L ML (*p* > 0.05). Although Acc L ML showed only substantial group differences when considering the average of three trials, if trials are considered, a group effect was evident (*p* < 0.05; reported in Fig. 3d). We identified lower values of SPARC in V, ML and AP axes, likely indicating greater movement intermittency and consequently less smoothness when individuals with PD performed the TUG test. Additionally, subjects with PD spent more time to complete the TUG test when compared to healthy controls (*p* < 0.05). Real-time visual inspections indicated that only a few participants showed freezing events during the test. Indeed, a more in-depth signal analysis showed moderate FR for PD subjects in our study (≈0.3) – indicating that only a few individuals showed pronounced freeze of gait during the task (Fig. 1k).

**Table 2:** Smoothness measures

		Subjects with PD	Healthy Controls	Effect Size & *p*-value
		*n* = 31	*n* = 6	PD vs HC
SPARC	Acc L total	−6.34 (− 6.98, − 5.70)	−4.23 (− 5.30, − 3.15)	1.52 (0.007*)
	Acc L V	− 5.07 (− 5.93, − 4.21)	−2.68 (− 3.37, − 1.99)	1.59 (0.019*)
	Acc L ML	−5.80 (− 6.30, − 5.30)	− 4.84 (− 6.00, − 3.67)	0.77 (0.115)
	Acc L AP	−3.25 (− 3.63, − 2.88)	−2.35 (− 2.69, − 2.00)	1.33 (0.041*)
	Acc A total	−4.99 (− 5.59, − 4.39)	−3.05 (− 3.37, − 2.73)	1.99 (0.007*)
	Acc A V	− 3.16 (− 3.60, − 2.72)	−2.14 (− 2.37, − 1.91)	1.44 (0.046*)
	Acc A ML	−4.52 (− 5.17, − 3.86)	−2.90 (− 3.17, − 2.62)	1.58 (0.035*)
	Acc A AP	−4.28 (− 4.81, − 3.75)	−2.90 (− 3.35, − 2.45)	1.46 (0.028*)
TUG time (s)		14.04 (12.34, 15.74)	9.15 (7.83, 10.47)	1.66 (0.016*)

Data are mean and 95% confidence interval

Abbreviations*. PD* Parkinson Disease, *HC* Healthy controls, *Acc L* linear acceleration, *Acc A* angular acceleration, *V* vertical, *ML* mediolateral, *AP* anteroposterior

*p < 0.05

**Fig. 2:**
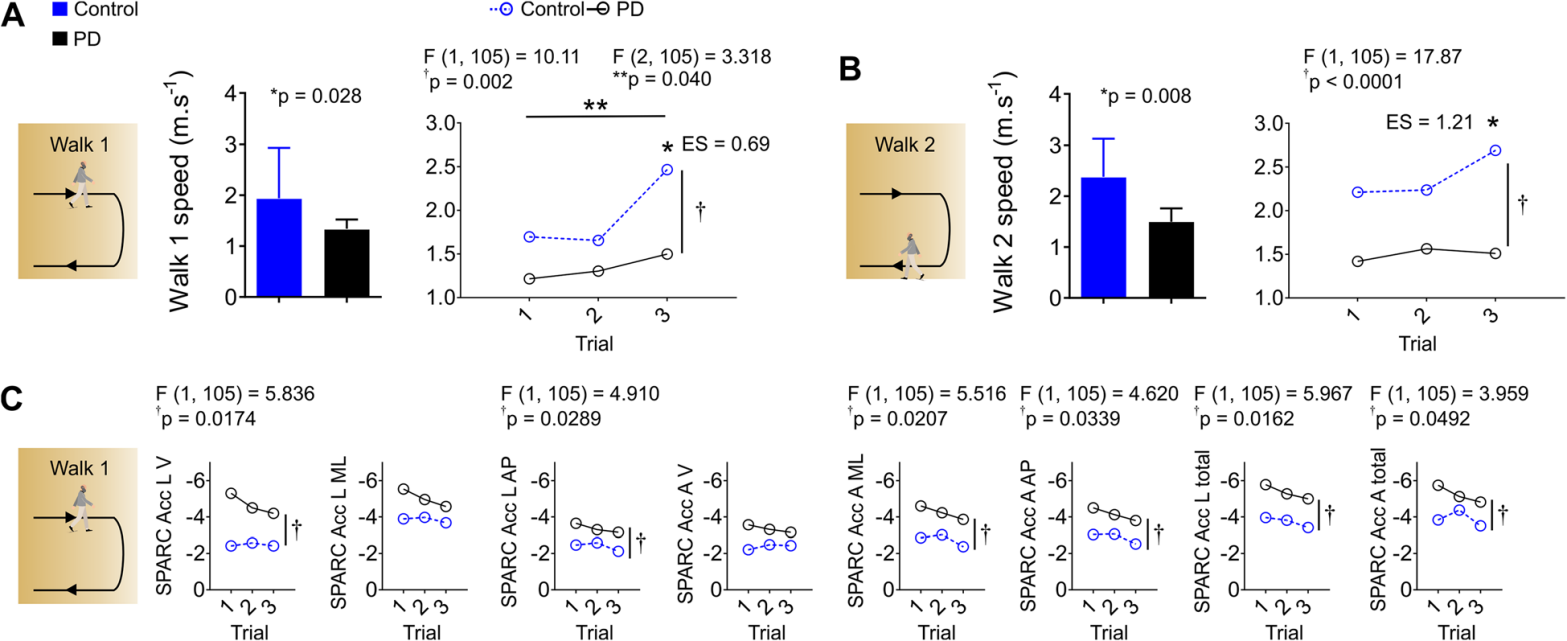
Walking speed increased between trials, but SPARC outcomes were stable across trials and not influenced by speed. **a**, **b** Walk 1 phase showed increased speed between trials, likely reflecting learning of the task. Both walking phases were slower for subjects with PD. **c** SPARC outcomes during the walk 1 phase showed absence of trial effects, suggesting independence of walking speed. Data is mean ± confidence interval 95% in **a** and **b** (middle panels) and Mean (other panels); n_Control_ = 6; n_PD_ = 31; *p < 0.05

To test if some learning occurred from the first to the third trial, we performed two-way ANOVAs using trial and group as factors. Both walking speeds (walk 1 and walk 2) were analyzed. A two-way ANOVA indicated a significant group effect and trial effect (*p* < 0.05, Fig. 2a, b). Post-hoc comparisons indicated that walking speed increased across trials, where healthy controls walked faster at the third trial compared to PD subjects. On the other hand, two-way ANOVA revealed only a significant group effect (*p* < 0.05) in the SPARC metrics without differences across trials (*p* > 0.05, Fig. 2c) for both walking bouts analyzed. This result suggests that SPARC was not susceptible to speed alterations. SPARC metrics from linear (Acc L) and angular accelerations (Acc A) were significantly lower in subjects with PD compared to healthy controls (*p* < 0.05).

**Fig. 3:**
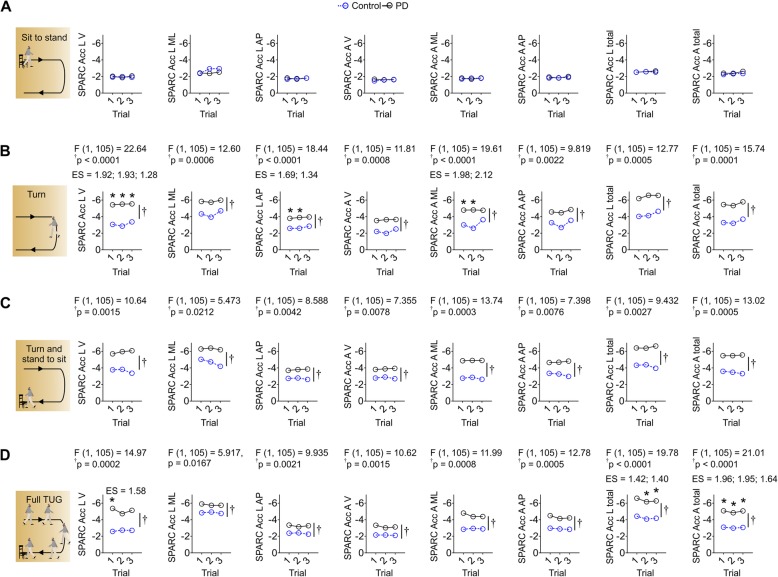
Turn was the most affected phase of TUG in subjects with PD and FOG. **a** Sit to stand showed lack of group effects. **b** Turning showed reduced SPARC mostly in the Acc L V, Acc L AP and Acc A ML components - these results are stronger at the first two trials. **c** Moderate group effects without post-hoc effects were evident during the stand to sit phase. **d** Full TUG showed reduced SPARC, mostly of Acc L total and Acc A total. Data is mean in all panels; n_Control_ = 6; n_PD_ = 31; *p < 0.05

While SPARC metrics did not show a significant difference across trials, there was a significant difference between groups for two phases of TUG – turn and turn and stand-to-sit – and also for full TUG test. There were no differences between groups for SPARC during the sit-to-stand phase of TUG (Fig. 3).

**Fig. 4:**
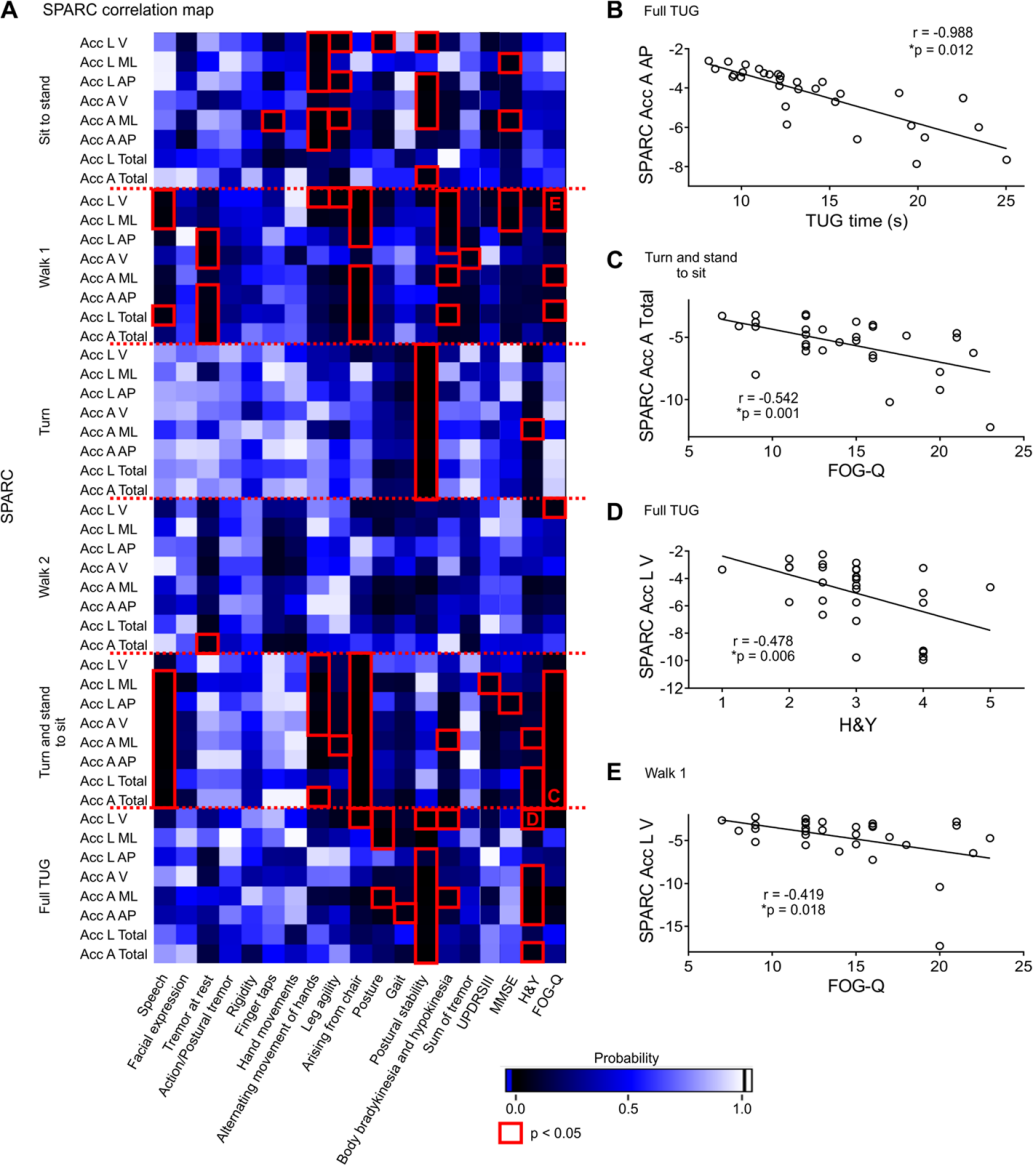
Correlation map between SPARC metrics and questionnaires. **a** Most pronounced correlations occurred during sit to stand, walk 1, turn and stand to sit phases. Briefly, the turn phase showed consistent correlations with postural stability scores; FOG-Q showed consistent correlations with walk 1 and stand to sit TUG phases; and full TUG correlated consistently with postural instability and H&Y scores, but partially with posture, bradykinesia and hyperkinesia scores. **b** SPARC Acc A AP correlated with full TUG duration. **c-e** Graphs of selected correlations – red letters in **a** (see Table 3 for full correlation data). n_PD_ = 31; red rectangles in **a** are *p* < 0.05; *p < 0.05

Correlations between SPARC values and clinical parameters are shown in Table 3 and Fig. 4. We investigated different parameters of UPDRS III on SPARC metrics. Some items of the UPDRS III, especially those related to arising from chair and postural stability, were moderately correlated with most of SPARC metrics in full TUG and two specific phases: (1) turn and (2) turn and stand to sit (*p* < 0.05). Also, we found consistent correlations between these two TUG phases with FOG and H&Y. Several other parameters presented a weak correlation with SPARC values as tremor at rest, finger taps, leg agility, gait, and posture.

**Table 3:** Correlations between smoothness (SPARC) and questionnaires

Questionnaires	TUG phase	SPARC variable	r	r^2^	*p*
Speech (from UPDRS III)	Turn and stand to sit	SPARC Acc A total	− 0.5312	0.2822	0.0021
	Turn and stand to sit	SPARC Acc L ML	−0.4927	0.2428	0.0049
	Turn and stand to sit	SPARC Acc A AP	−0.4832	0.2335	0.0059
	Turn and stand to sit	SPARC Acc A V	−0.4782	0.2287	0.0065
	Turn and stand to sit	SPARC Acc L AP	−0.4718	0.2226	0.0074
	Turn and stand to sit	SPARC Acc A ML	−0.4616	0.2131	0.0089
	Turn and stand to sit	SPARC Acc L total	−0.4239	0.1797	0.0175
	Walk 1	SPARC Acc L ML	−0.4216	0.1777	0.0182
	Walk 1	SPARC Acc L V	−0.3872	0.1500	0.0314
	Walk 1	SPARC Acc L total	−0.3857	0.1488	0.0321
Tremor at Rest (from UPDRS III)	Walk 1	SPARC Acc A V	0.3990	0.1592	0.0262
	Walk 1	SPARC Acc A total	0.3873	0.1500	0.0314
	Walk 1	SPARC Acc L AP	0.3648	0.1331	0.0436
	Walk 1	SPARC Acc L total	0.3643	0.1327	0.0439
	Walk 2	SPARC Acc A total	0.3596	0.1293	0.0469
	Walk 1	SPARC Acc A AP	0.3563	0.1269	0.0491
Finger Taps (from UPDRS III)	Sit to stand	SPARC Acc A ML	−0.3598	0.1294	0.0468
Alternating Movements of Hands (from UPDRS III)	Sit to stand	SPARC Acc A AP	−0.4761	0.2267	0.0068
	Turn and stand to sit	SPARC Acc L ML	−0.4281	0.1833	0.0163
	Sit to stand	SPARC Acc A ML	−0.4273	0.1825	0.0165
	Turn and stand to sit	SPARC Acc A AP	−0.4133	0.1708	0.0208
	Sit to stand	SPARC Acc L V	−0.4124	0.1701	0.0211
	Turn and stand to sit	SPARC Acc A V	−0.4023	0.1618	0.0249
	Turn and stand to sit	SPARC Acc A total	−0.3982	0.1586	0.0265
	Turn and stand to sit	SPARC Acc A ML	−0.3887	0.1511	0.0307
	Sit to stand	SPARC Acc L ML	−0.3778	0.1427	0.0361
	Sit to stand	SPARC Acc L AP	−0.3707	0.1374	0.0401
	Walk 1	SPARC Acc L V	− 0.3671	0.1347	0.0422
	Turn and stand to sit	SPARC Acc L AP	−0.3565	0.1271	0.0490
Leg Agility (from UPDRS III)	Walk 1	SPARC Acc L V	−0.4387	0.1925	0.0136
	Sit to stand	SPARC Acc A ML	−0.4090	0.1672	0.0224
	Sit to stand	SPARC Acc L V	−0.3867	0.1495	0.0316
	Turn and stand to sit	SPARC Acc A ML	−0.3654	0.1335	0.0433
	Sit to stand	SPARC Acc L AP	−0.3566	0.1272	0.0489
Arising from Chair (from UPDRS III)	Turn and stand to sit	SPARC Acc A total	−0.6233	0.3885	0.0002
	Turn and stand to sit	SPARC Acc A AP	−0.6098	0.3718	0.0003
	Turn and stand to sit	SPARC Acc A ML	−0.6054	0.3666	0.0003
	Walk 1	SPARC Acc L total	−0.5838	0.3408	0.0006
	Walk 1	SPARC Acc L V	−0.5680	0.3227	0.0009
	Turn and stand to sit	SPARC Acc A V	−0.5570	0.3102	0.0011
	Walk 1	SPARC Acc L ML	−0.5385	0.2900	0.0018
	Turn and stand to sit	SPARC Acc L AP	−0.5107	0.2608	0.0033
	Turn and stand to sit	SPARC Acc L total	−0.5065	0.2565	0.0036
	Full TUG	SPARC Acc L V	−0.4878	0.2380	0.0054
	Walk 1	SPARC Acc A ML	−0.4577	0.2095	0.0096
	Turn and stand to sit	SPARC Acc L ML	−0.4542	0.2063	0.0103
	Walk 1	SPARC Acc A AP	−0.4487	0.2013	0.0113
	Turn and stand to sit	SPARC Acc L V	− 0.4471	0.1999	0.0117
	Walk 1	SPARC Acc L AP	−0.3663	0.1342	0.0427
	Walk 1	SPARC Acc A total	−0.3612	0.1305	0.0459
Posture (from UPDRS III)	Full TUG	SPARC Acc L ML	−0.4779	0.2284	0.0065
	Full TUG	SPARC Acc A ML	−0.3838	0.1473	0.0330
	Sit to stand	SPARC Acc L V	−0.3710	0.1376	0.0399
	Full TUG	SPARC Acc L V	−0.3652	0.1334	0.0434
Gait (from UPDRS III)	Full TUG	SPARC Acc A AP	−0.3617	0.1308	0.0456
Postural stability (from UPDRS III)	Turn	SPARC Acc L total	−0.6492	0.4215	0.0001
	Turn	SPARC Acc L V	−0.6424	0.4127	0.0001
	Turn	SPARC Acc L AP	−0.6270	0.3932	0.0002
	Turn	SPARC Acc A ML	−0.5975	0.3570	0.0004
	Turn	SPARC Acc L ML	−0.5498	0.3023	0.0014
	Full TUG	SPARC Acc L ML	− 0.5497	0.3022	0.0014
	Full TUG	SPARC Acc A V	−0.5353	0.2865	0.0019
	Sit to stand	SPARC Acc L AP	−0.5231	0.2736	0.0025
	Turn	SPARC Acc A total	−0.5186	0.2689	0.0028
	Full TUG	SPARC Acc L V	−0.4911	0.2412	0.0050
	Full TUG	SPARC Acc A total	−0.4865	0.2367	0.0055
	Full TUG	SPARC Acc A ML	−0.4832	0.2335	0.0059
	Turn	SPARC Acc A V	−0.4730	0.2237	0.0072
	Full TUG	SPARC Acc A AP	−0.4525	0.2047	0.0106
	Sit to stand	SPARC Acc A ML	−0.4486	0.2012	0.0114
	Turn	SPARC Acc A AP	−0.4343	0.1886	0.0146
	Sit to stand	SPARC Acc L V	−0.4306	0.1855	0.0156
	Full TUG	SPARC Acc L total	−0.4272	0.1825	0.0165
	Sit to stand	SPARC Acc A V	−0.4130	0.1706	0.0209
	Sit to stand	SPARC Acc A total	−0.3803	0.1446	0.0348
Body bradykinesia and hypokinesia (from UPDRS III)	Walk 1	SPARC Acc L V	−0.4633	0.2146	0.0087
	Walk 1	SPARC Acc L total	−0.4499	0.2024	0.0111
	Walk 1	SPARC Acc L ML	−0.4092	0.1674	0.0223
	Full TUG	SPARC Acc L V	−0.4020	0.1616	0.0250
	Walk 1	SPARC Acc A ML	−0.3877	0.1503	0.0312
	Walk 1	SPARC Acc L AP	−0.3804	0.1447	0.0348
	Full TUG	SPARC Acc A ML	−0.3746	0.1403	0.0379
	Turn and stand to sit	SPARC Acc A ML	−0.3701	0.1370	0.0404
Sum of Tremor (from UPDRS III)	Walk 1	SPARC Acc A V	0.3583	0.1284	0.0478
UPDRS III	Turn and stand to sit	SPARC Acc L ML	−0.3557	0.1265	0.0495
MMSE	Walk 1	SPARC Acc L ML	0.3951	0.1561	0.0278
	Sit to stand	SPARC Acc L ML	0.3802	0.1445	0.0349
	Turn and stand to sit	SPARC Acc L AP	0.3642	0.1326	0.0440
	Sit to stand	SPARC Acc A ML	0.3583	0.1284	0.0478
	Walk 1	SPARC Acc L V	0.3556	0.1265	0.0496
H&Y	Full TUG	SPARC Acc L V	−0.4786	0.2291	0.0065
	Full TUG	SPARC Acc A ML	−0.4623	0.2137	0.0088
	Full TUG	SPARC Acc A AP	−0.4527	0.2050	0.0105
	Full TUG	SPARC Acc A total	−0.3775	0.1425	0.0363
	Turn and stand to sit	SPARC Acc L total	−0.3687	0.1359	0.0413
	Turn	SPARC Acc A ML	−0.3680	0.1355	0.0416
	Turn and stand to sit	SPARC Acc A ML	−0.3616	0.1307	0.0457
	Full TUG	SPARC Acc A V	−0.3581	0.1282	0.0479
	Turn and stand to sit	SPARC Acc A total	−0.3580	0.1281	0.0480
FOG-Q	Turn and stand to sit	SPARC Acc A total	−0.5420	0.2938	0.0016
	Turn and stand to sit	SPARC Acc A ML	−0.5302	0.2811	0.0022
	Turn and stand to sit	SPARC Acc A AP	−0.5203	0.2707	0.0027
	Turn and stand to sit	SPARC Acc A V	−0.5110	0.2612	0.0033
	Turn and stand to sit	SPARC Acc L AP	−0.5096	0.2597	0.0034
	Turn and stand to sit	SPARC Acc L total	−0.4538	0.2059	0.0104
	Full TUG	SPARC Acc A ML	−0.4398	0.1934	0.0133
	Turn and stand to sit	SPARC Acc L ML	−0.4336	0.1880	0.0148
	Walk 1	SPARC Acc L V	−0.4197	0.1761	0.0188
	Full TUG	SPARC Acc L V	−0.4188	0.1754	0.0190
	Walk 1	SPARC Acc L ML	−0.4079	0.1664	0.0227
	Walk 1	SPARC Acc L total	−0.4009	0.1607	0.0254
	Walk 2	SPARC Acc L V	−0.3752	0.1408	0.0375
	Walk 1	SPARC Acc A ML	−0.3735	0.1395	0.0385

Abbreviations**.** *Acc L* linear acceleration, *Acc A* angular acceleration, *V* vertical, *ML* mediolateral, *AP* anteroposterior, *PD* Parkinson Disease, *MMSE* Mini Mental State Examination, *H&* Hoehn & Yahr scale, *UPDRS III* motor part of the Unified Parkinson’s Disease Rating Scale, *FOG-Q* Freezing of Gait Questionnaire, *TUG* Timed up and Go test

Note. Pearson’s correlations (*p* < 0.05)

## Discussion

In this study, we report the quantification of movement smoothness (i.e., SPARC) during a functional mobility test using an IMU system. Individuals with PD and FOG were evaluated in the off-medication phase and data were compared to healthy controls. Subjects with PD showed abnormal spectral-profile of oscillatory movements performed during the TUG test, likely reflecting subtle FOG effects over SPARC metrics. It means that subjects with PD and FOG presented higher movement intermittency and less smoothness during the functional mobility task (TUG), which were correlated with clinical parameters.

Our results suggest that some learning occurred from the first to the third TUG trials, and this may affect the duration and variability outcomes of TUG. Conversely, SPARC was not affected by speed, in line with previous recommendations, we suggest this is an important advantage of using the SPARC metrics to evaluate the TUG task. Standard measures of duration are susceptible to this type of learning of the task and may bias outcomes. Measurements such dimensionless jerk and SPARC show reduced effects of movement amplitude and duration, in addition and as aforementioned, SPARC is less susceptible to noise artifacts [13].

Previous research quantified SPARC metrics during a walking-only task in subjects with PD without FOG [19]. We corroborate their findings using a more complex task, which involves turns, brakes, sit-to-stand and stand-to-sit movements. Also, motor symptoms related to slowness, arrhythmicity, and fragmentation of movement may express differently during gait-only trials and complex test trials, i.e., TUG [14, 19]. Our analysis extends the possibilities of using the TUG test by adding important information about variables related to mobility smoothness.

The outcomes of functional mobility and risk of falls are measured by the total time to perform the TUG task - cut-off score of 11.5 s to discriminate PD fallers and non-fallers [9]. However, subjects with PD can show good walking speed and complete the TUG task fast - with a normal TUG duration - but present poor quality performance [34]. Albeit, many times such individuals show noticeable deficits during the task – which could indicate impaired mobility and risk of falls. In these cases, the simple duration of TUG is not sensitive to capture such impairments. Previous studies quantified movement quality (smoothness) during the iTUG test using Jerk analysis [30, 31]. Subjects with PD and FOG have more gait difficulties while performing the TUG and while performing turning tasks in daily life with reduced movement quality [30, 31]. This task is more challenging for subjects with PD and FOG – when compared with those without [30, 31]. Importantly, turning while walking is related to FOG and falls [35]. In general, subjects with PD turn with reduced trunk dissociation (“turn en bloc”), slower and shorter steps [35, 36]. In our results, subjects with PD and FOG presented reduced smoothness especially in the turn phase of TUG, when compared to healthy controls. Our results corroborate with previous findings and add a new innovative approach for detecting smoothness (SPARC) during the TUG task.

We also reported poor smoothness when subjects with PD were in transition from turning and stand to sit. Strategies while performing these tasks were investigated in individuals with PD, and it was shown that they present difficulties to sequence two motor tasks at the same time, such as turning and sitting [35]. Authors suggest that this difficulty to sequential and complex motor tasks is due to reduced rotation of body segments, reduced balance control, and diminished ability to adequate eccentric muscle contractions of lower limbs [35]. This strategy is not possible to measure in detail using only the TUG duration. Smoothness analysis, such as the SPARC using IMU, is sensible to detect such movement alterations that require turns – especially due to the high sensitivity of accelerometers and gyroscopes.

Moreover, we found some moderate correlations between questionnaires and SPARC values in three specific cases: (1) full TUG, (2) turn and stand to sit, and (3) turn. Interestingly, most of the important correlations involved turn, which is very challenging in freezers [6, 30, 31, 37]. Subjects with PD and FOG need a greater effort to regulate cadence, stride length variability, speed, and trunk motion during turn conditions (when compared to straight walk) [34, 38]. We found more pronounced differences between groups on SPARC metrics when turn was involved. SPARC metrics in these situations were related to FOG and disease severity (H&Y stage). Also, two items of UPDRS III related to arising from chair and postural instability were correlated moderately with most of SPARC metrics. Interesting, the ability to arise from a chair lacked correlation with unsmooth movements during sit to stand, but showed abundant correlations with the subsequent walking bout and with the ability to smoothly turn and sit on the chair. Overall, it seems that smoothness is impaired when participants with PD and FOG need to perform challenging tasks, such as when preparing sharp turns while walking or to turn and sit on a chair. These two UPDRS III items were related to motor fluctuations and axial function during gait in subjects with PD [39]. Also, postural instability is substantially impaired in individuals with FOG [38]. Trunk rigidity hampers trunk dissociation between head, thoracic and pelvic girdle to perform turns during the TUG test, and this problem could influence smoothness. Turn assessment is mandatory to identify postural instability and could be easily implemented into the commercial IMU software to report SPARC values in real-time and at a low computational cost [34]. We suggest that smoothness (SPARC) during the TUG test may be adjuvant to indicate poor functional mobility quality, FOG, disease severity and symptoms like postural instability. These symptoms added to compensatory strategies can release some problems related to movement quality as above mentioned. These problems are quite difficult to measure in clinical practice because it is subjective and may change between therapists, making it difficult to measure motor quality improvements before and after rehabilitation programs.

Our results show the first evidence of SPARC outcomes during the iTUG task in subjects with FOG. Here, we describe normative values for the full TUG task as ≈ − 3.2 (Control) and − 4.7 (PD); specifically, for walk 1 of ≈ − 3.1 (Control) and − 4.4 (PD), for turn of ≈ − 3.3 (Control) and − 5.0 (PD) and for walk 2 of ≈ − 2.5 (Control) and − 4.4 (PD). If contrasted with previous reports of SPARC outcomes during a different task (i.e., long gait-only task: 50 m walk; ≈ 40s of duration) our results are overall smaller [19]. This reflects the SPARC task sensitivity between point-to-point reaching (≈ − 1.6) [13], 50 m walking (≈ − 5.5) [19] and TUG tasks (≈ − 3.2).

Limitations of the present study include a lack of PD group without FOG to investigate FOG contribution to SPARC outcome. Additionally, we did not perform a synchronized video analysis to confirm FOG episodes as indicated by the SPARC analysis. Future studies should evaluate not only the differences between FOG+ and FOG- groups based on questionnaires but also using machine learning-based detections of the amount of FOG during the task [28, 29]. This methodology would provide a more straightforward link between the specific amount of FOG and SPARC. Another limitation is the reduced number of steps during the TUG performance, which, especially for healthy controls, is not optimum for analyzing gait variability [40]. Artificially increasing data segments and the SPARC ability to solve duration issues [13] were solutions used in the present study to overcome this limitation. Additional work is also needed to explore SPARC reference values that could be easily applied in the clinical practice and it may offer a more refined scale for assessing fall risk.

## Conclusions

Movement quality evaluation, such as smoothness, may be a better outcome measurement for the detection of motor impairments in subjects with PD who show a “normal” TUG time. In summary, our results support the use of IMUs for SPARC smoothness calculations of individuals with PD and FOG. This quantitative measurement of multi-functional domains of TUG test should provide a more objective and detailed description of functional mobility. Our results confirmed our hypothesis: subjects with PD and FOG present less smoothness during functional mobility assessment (TUG test) and these data are related to clinical parameters. The most striking findings of this study indicate that subjects with PD and FOG display remarkable unsmooth movements when turning while walking. Further clinical trials should also include a PD group without FOG in the analysis and investigate all parts of the TUG test, including turning movements. Assessment of movement quality during the TUG test may be important to guide the clinical treatment of individuals with PD. Future studies should detect FOG epochs, categorize FOG events in subtypes and relate such events with SPARC metrics. A better understanding of the specific contribution of FOG to SPARC may allow the construction of finer-grained assessments, medical population classification, and fall risk prediction.

## Additional file

Additional file 1: Algorithm to calculate SPARC from IMU system. (DOCX 15 kb)

## Data Availability

Not applicable.

## Abbreviations

Acc A: Angular acceleration
Acc L: Linear acceleration
AP: Anteroposterior
DC: Direct current
FOG: Freezing of gait
H&Y: Hoehn & Yahr scale
IMUs: Inertial Measurement Units
ML: Mediolateral
MMSE: Mini Mental State Examination
PD: Parkinson’s disease
SPARC: Spectral arc length
TUG: Timed Up and Go
UPDRS III: Motor part of the Unified Parkinson’s Disease Rating Scale
V: Vertical
