# Supplementary material for: Movement smoothness during a functional mobility task in subjects with Parkinson’s disease and freezing of gait – an analysis using inertial measurement units
Source: J Neuroeng Rehabil. 2019 Sep 5;16:110. doi: 10.1186/s12984-019-0579-8 (PMC6729092; doi:10.1186/s12984-019-0579-8)
Supplement: Supplementary file 1 — Algorithm to calculate SPARC from IMU system. (DOCX 15 kb) [file 12984_2019_579_MOESM1_ESM.docx]

**Additional file 1**

Algorithm to calculate SPARC from IMU system

Step 1: open .txt file with the acceleration data;

Step 2: remove low frequency DC using mean subtractions;

Step 3: artificially increase data segments of each TUG phase by a factor of 4 (smaller TUG phases: sit to stand, walk 1, turn, walk 2 and stand to sit) and 2 (full TUG) and apply zero padding;

Step 4: calculate the Power Spectrum Density (PSD), and normalize PSD by the peak value to calculate *normPSD*;

Step 5: crop step 4 waveform to apply the limits of integration – i.e., use only the Fourier spectrum from 0 Hz to 10 Hz;

Step 6: use 1 divided by 10 as a magnitude adjustment constant;

Step 7: squared steps 5 and 6 and sum;

Step 8: square root step 7;

Step 9: numeric integration of step 8;

Step 10: multiply step 9 by -1.
